# Digitial memory test for detecting and assessing cognitive impairment in parkinson's disease

**DOI:** 10.1002/alz.088469

**Published:** 2025-01-09

**Authors:** Zhong Pei

**Affiliations:** ^1^ The First Affiliated Hospital, Sun Yat‐sen Univesrity, Guangzhou, Guangdong China

## Abstract

**Background:**

The objectives of the study is to assess the clinical utility of the MemTrax Memory Test for detection of cognitive impairment in patients with PD.

**Method:**

The MemTrax and Montreal Cognitive Assessment (MoCA) were administered to 61 healthy controls (HC), 102 PD patients with normal cognition (PD‐N), 74 PD patients with mild cognitive impairment (PD‐MCI) and 52 PD patients with dementia (PD‐D). The MemTrax performance, MTx‐%C, MTx‐RT and MTx‐Cp, and the MoCA scores were comparatively analyzed. The MemTrax performances were assessed according to the areas under the receiver operating characteristic curve.

**Result:**

The MoCA scores were similar between HC and PD‐N, however, MTx‐%C and MTx‐Cp were lower in PD‐N than HC(p<0.05). MTx‐%C, MTx‐Cp and the MoCA scores were significantly lower in PD‐MCI versus PD‐N and in PD‐D versus PD‐MCI (p ≤ 0.001), while MTx‐RT was statistically longer in PD‐D versus PD‐MCI (p ≤ 0.001). For the PD groups, the MemTrax performance strongly correlated with the MoCA scores. To detect PD‐MCI, the optimal MTx‐%C and MTx‐Cp cutoff were 75% and 50.0, respectively. To detect PD‐D, the optimal MTx‐%C, MTx‐RT and MTx‐Cp cutoff were 69%, 1.341s and 40.6, respectively.

**Conclusion:**

The MemTrax provides rapid, valid and reliable metrics for assessing cognition in patients with PD that could have practical clinical utility for identifying PD‐MCI at early stage and monitoring cognitive function decline during the progression of disease.

